# Intersectoral collaboration for supporting the health and wellbeing of Aboriginal families and children in out-of-home care: perspectives from Western Australian Aboriginal Community Controlled Health Organisations

**DOI:** 10.3389/fpubh.2024.1392208

**Published:** 2024-06-25

**Authors:** Sasha Moodie, Jocelyn Jones, Richard Chenhall, Robyn Williams, Cathy Garlett, Alison Gibberd, Melissa O’Donnell, Dan McAullay, Bridgette McNamara, Sandra Eades

**Affiliations:** ^1^National Drug Research Institute, Curtin University, Perth, WA, Australia; ^2^Melbourne School of Population and Global Health, The University of Melbourne, Melbourne, VIC, Australia; ^3^Curtin Medical School, Faculty of Health Sciences, Curtin University, Bentley, WA, Australia; ^4^Hunter Research Medical Institute, Newcastle, NSW, Australia; ^5^Australian Centre for Child Protection, University of South Australia, Adelaide, SA, Australia; ^6^Kurongkurl Katitjin, Edith Cowan University, Perth, WA, Australia; ^7^Barwon South West Public Health Unit, Barwon Health, Geelong, VIC, Australia

**Keywords:** Aboriginal and Torres Strait Islander, out-of-home care, primary health care, Australia, kinship care, intersectoral collaboration, Indigenous

## Abstract

**Introduction:**

Western Australia has one of the highest rates of Aboriginal children entering out-of-home care in Australia. Kinship care is the preferred culturally safe out-of-home care option for Aboriginal children, yet all jurisdictions, including Western Australia, are far from meeting best-practice national standards. Intersectoral collaboration is a key primary healthcare principle and internationally recognized for improving health systems and outcomes. This paper presents findings from a qualitative research project investigating Aboriginal primary healthcare workers’ experiences of intersectoral collaboration challenges and strengthening opportunities.

**Methods:**

Constructivist grounded theory guided this research involving 55 semi-structured interviews and four focus group discussions with Aboriginal primary healthcare workers. The research was guided by Indigenous methodologies and led by Indigenous researchers Participants were recruited from seven Aboriginal Community Controlled Health Organisations located across Perth metro, Pilbara, Midwest/Gascoyne and Southwest regions in Western Australia.

**Results:**

Key themes identified around intersectoral collaboration challenges were communication, including information sharing and interagency meetings, and the relationship with the government sector, including trust and the importance of the perception of Aboriginal health service independence. Key themes around strengthening areas to improve intersectoral collaboration included strengthening service resourcing and coverage, including the availability of services, and addressing high program turnover. The need for a shift in approach, including more emphasis on Aboriginal-led care and aligning approaches between sectors, was another area for strengthening.

**Discussion:**

This study addresses a significant research gap concerning out-of-home care, kinship care, and intersectoral collaboration in an Australian Aboriginal context. Findings highlighted the need to review the out-of-home and kinship models of care to strengthen the system, including creating more formal and structured modes of collaborating and better resourcing family support and kinship care.

## 1 Introduction

Government assimilation policies implemented between 1910 and 1970 resulted in the forced removal of Aboriginal and Torres Strait Islander children from their families. Known as the ‘Stolen Generations’, these removals had a profound and harmful impact on Aboriginal and Torres Strait Islander people’s (respectfully referred to as Aboriginal people from hereon) social, cultural and emotional wellbeing (1, 2). The repercussions of these detrimental policies have led to enduring effects, with intergenerational trauma manifesting and exacerbating adverse social and health outcomes among Aboriginal communities and populations (2, 3). The lasting effects of colonization and modern experiences of institutional racism, socioeconomic disadvantage, lack of housing, and poverty have been found to be contributing social determinants of health that present challenges for Aboriginal parents in providing care for their children (4, 5). International bodies have denounced the long history of injustices experienced by Australian Aboriginal people and Indigenous populations globally (6).

The Department of Communities within the Western Australian (WA) state government has the statutory authority under the Children and Community Services Act (2004) to respond to reports of child safety and welfare concerns (7). Under the Act, a child is considered to be in need of protection if they have suffered, or are at risk of suffering, abuse (physical, emotional and/or sexual), neglect and the child’s parents have not, or cannot, protect them from harm (7, 8).

Aboriginal children are overrepresented at all stages of the child protection system in Australia. This includes from notification to child protection services, investigation of notification, substantiation of notification (reasonable cause found for child welfare concerns) and placement of children in out-of-home care (9). The rising rate of Aboriginal children entering out-of-home care (children being removed from their families by state government child protection services) in Australia is increasingly being referred to as a ‘second stolen generation’ by local community members and risks perpetuating the adverse impacts on health and wellbeing linked to the institutional removal of Aboriginal children from their families, communities, and culture (3, 10).

### 1.1 Out-of-home care

Out-of-home care is defined as ‘overnight care for children under 18 who are unable to live with their families due to child safety concerns’ (11). Nationally, Aboriginal children continue to be overrepresented in out-of-home care, with 56.8 per 1,000 Aboriginal children in out-of-home care compared to 4.8 per 1,000 non-Aboriginal children (almost 12 times the rate) according to 2021–2022 data (12). In WA, the rate of Aboriginal children in out-of-home care is higher than the national average, with 61.6 per 1,000 Aboriginal children in out-of-home care compared to 3.1 per 1,000 non-Aboriginal children (20 times the rate) (12). The disproportionate rate at which Aboriginal children are being removed from their families compared with non-Aboriginal children is hugely problematic, especially considering the long history of mistreatment and harmful government policies toward Aboriginal people and populations in Australia.

### 1.2 What is kinship care?

For Aboriginal people, a kinship carer may be ‘another Indigenous person who is a member of their community, a compatible community, or from the same language group’ (13). Kinship care may either be formal or informal. Formal kinship carers can access available support services, including financial support from their state or territory, for caring for a child (14). Informal kinship carers, who are not formally acknowledged in the out-of-home care system, are not able to access state-based out-of-home care support services (14). Kinship care is not a new concept for Aboriginal people and populations. There is a longstanding tradition of community-based care in Aboriginal culture with immediate family, children and extended family (blood related and non-blood related) all having a role in raising a child (15). Because of this long-standing cultural practice, when Aboriginal parents need support to look after their child, a child’s kin often feel a sense of cultural obligation to ensure the child continues to be raised in their community and culture (15–17).

### 1.3 Kinship care and Aboriginal children in out-of-home care

The Australian Aboriginal and Torres Strait Islander Child Placement Principle (ATSICPP) acknowledges the significance of family, community, culture, and country in child and family welfare laws, policies and practices (18). The ATSICPP was established in 1984 after a prolonged advocacy campaign led by Aboriginal and Torres Strait Islander peoples, Aboriginal Community Controlled Organisations (ACCOs), and other groups in response to the increasing number of Aboriginal children placed in out-of-home care with non-Aboriginal carers (18, 19). This framework maintains that if an Aboriginal child is to be placed in out-of-home care, the following care options should be prioritised (in order): (a) relatives or kin; (b) other Aboriginal carers from the child’s community; and (c) Aboriginal carers from another community (20). While the rate of Aboriginal children placed in out-of-home care is a trend that continues to rise, the proportion of children placed with kinship carers is declining (5, 21–24). There is no conclusive evidence on why the rate of kinship carers is declining; however, this is likely the result of children in informal kinship care arrangements being unmonitored and the pressure on the finite number of kinship carers (24–27). Despite the important role of kinship carers, evidence suggests kinship carers are significantly unsupported and undervalued (14, 21, 28).

### 1.4 The role of Aboriginal Community Controlled Health Organisations (ACCHOs)

For Aboriginal people, health is considered to be ‘not just the physical well-being of the individual, but the social, emotional, and cultural well-being of the whole community’ (29). This is a whole-of-life approach to health and considers the cyclical concept of life and death (29). Aboriginal Community Controlled Health Organisations (ACCHOs) are not-for-profit organisations that are controlled and governed by Aboriginal people and connected to the communities in which they provide services (30). Guided by Aboriginal definitions and perspectives on health, ACCHOs are healthcare services established and governed by the local Aboriginal community with the purpose of providing holistic, culturally appropriate, and comprehensive healthcare to the community that governs it (31). ACCHOs were set up to address the unmet health needs of Aboriginal clients, including advocacy, in response to poor engagement of Aboriginal people in mainstream health services (32). In Aboriginal communities, the primary healthcare provided by ACCHOs is ‘based on practical, scientifically sound, socially and culturally acceptable methods and technology made universally accessible to individuals and families in the communities in which they live through their full participation at every stage of development in the spirit of self-reliance and self-determination’ (29). Because ACCHOs are considered culturally safe and adopt a holistic model of care, they provide a range of different services to Aboriginal communities, including child protection, family, and kinship carer support services. To reduce the number of Aboriginal children in out-of-home care, the ATSICPP recommends greater involvement of ACCOs in the management of Aboriginal child protection matters in all jurisdictions (18). This requires collaboration between sectors – in particular, between Aboriginal organisations and government social services sectors.

### 1.5 Intersectoral collaboration

Intersectoral collaboration is a key primary healthcare principle and refers to ‘the collective actions involving more than one specialized agency, performing different roles for a common purpose’ (33). Intersectoral collaboration in healthcare recognizes the complexity of the social determinants of health and how the determinants intersect to influence health outcomes (34). It recognizes that different government, non-government, and community organisations, across health and non-health related sectors, must work together to achieve public health action through addressing social factors that act as contributors to adverse health and wellbeing outcomes (34). Despite the concepts often being used interchangeably, intersectoral collaboration differs from interagency collaboration as it emphasizes the meaningful action agencies from non-health sectors can contribute to public health improvement (35). Intersectoral approaches to addressing complex health problems have been increasingly promoted by international organisations and institutions, including the World Health Organisation, with concepts including ‘intersectoral action for health equity’ and ‘health in all policies’ becoming widely recognized and adopted in public health practice (36, 37). These approaches incentivise collaboration between health and non-health sectors by emphasizing a ‘win-win’ approach, whereby desirable outcomes are mutually beneficial for all sectors involved (35, 37). However, despite widespread recognition and acceptance of intersectoral approaches, there is a limited evidence base discussing how intersectoral collaboration can be transformed into action (38).

In Danaher’s (39) paper exploring the enablers and barriers to intersectoral collaboration, the authors identified the following enablers: strong partner relationships, having a shared vision, strong and equitable leadership, access to resources and working within a structured process-oriented model of care. Mutual trust and respect, fair decision-making processes, clear and effective communication and effective leadership were found to characterize successful intersectoral relationships (39). These factors promoting intersectoral collaboration have been replicated in recent literature, with common themes around access to resources, communication, trust, shared vision, decision-making equality, and structured and integrated modes of collaborating (36, 37, 39–44). Other studies have emphasized external influences that can have an impact on intersectoral collaboration, including the importance of sustainable financing and having access to secure, long-term and comprehensive financial means to sustaining intersectoral partnerships (45). Shifting social norms, community influences and political contexts have also been found to affect intersectoral collaboration (40).

There is no ‘one-size-fits-all model’ on how intersectoral collaboration should be approached (37). This is because context matters: what works in one setting may not in another (37). However, Kuruvilla et al.’s (46) study, which analysed case studies from widely differing social, economic, geographic, cultural and historical contexts, noted that despite the heterogeneity of their case studies, there were strong similarities identified in how different sectors collaborated.

### 1.6 Intersectoral collaboration: Aboriginal out-of-home care and kinship care

A systematic literature search produced limited results in relation to kinship care, child protection and intersectoral collaboration in an Australian context. Articles focusing on WA were particularly sparse. Because of this, this research paper drew from studies in broader primary healthcare and intersectoral collaboration settings. While there is research that addresses intersectoral collaboration in Aboriginal primary healthcare settings, studies tend to advocate for the importance of intersectoral collaboration within broader research scopes and do not directly address or interrogate how collaboration plays out (47–52). A recent study conducted by Osborn et al. (53) in a remote New South Wales community researched a variety of community-based healthcare services for Aboriginal people, including local ACCHOs. A key theme Osborn et al. (53) identified through their analysis was the under-servicing and over-servicing among a variety of Aboriginal healthcare services in the community, which provided insight into the ‘systemic barriers to interagency cooperation’ (53). These findings were attributed to poor resourcing and incentivisation for health providers to coordinate their services (53).

Similarly, a recent study on best-practice care for mothers and their Aboriginal babies in WA highlighted the need for strengthened relationships, collaboration, and communication across sectors to facilitate a best-practice standard of care (54). Another study focusing on dementia in a remote Indigenous community in the Northern Territory identified intersectoral collaboration and under-resourcing as key challenges and advocated for additional service delivery and support for carers (55). These research findings are in line with Anaf et al.’s (34) study, which explored factors that shape intersectoral collaboration and action among primary healthcare providers in an Australian context. While Anaf et al.’s study (34) involved six primary healthcare services in Australia, only two of the services were Aboriginal controlled, and none of the services were in WA, highlighting a limitation in the applicability of their overall results to the WA Aboriginal primary healthcare provider context. However, many of Anaf et al.’s (34) findings indicate alignment with broader research on intersectoral collaboration and their findings, including the importance of shared values, consistent approaches and access to financial and human resources, are consistent with research in Aboriginal primary healthcare settings (56).

While WA Government departments across sectors – including social services, housing, education and disability – rely on their intersectoral partnerships with ACCHOs to improve health-seeking behavior and bridge the communication gap between Aboriginal communities and government agencies, ACCHOs are not being appropriately supported to scale up and deliver their services (24, 57). In response to this criticism, the Department of Communities released the Aboriginal Community Controlled Organisation Strategy 2022–2032 (30). This strategy details the WA Government’s commitment to improve health outcomes for Aboriginal people and populations through better supporting ACCHOs to deliver culturally secure services (30). This renewed commitment was announced shortly after the 2021 Family Matters report found WA was tracking behind all jurisdictions on several targets (22). This included WA having the lowest proportional investment in family support services in the country (22). Intersectoral collaboration between stakeholders within the WA Government, non-government organisations and Aboriginal organisations, including ACCHOs, is critical to address the overrepresentation of Aboriginal children in out-of-home care and the health and wellbeing of Aboriginal kinship carers (31, 52, 56, 58). Despite the recognition that intersectoral collaboration is important in public health practice, there is no specific evidence addressing intersectoral collaboration in the context of out-of-home care and kinship care in an Australian Aboriginal context (53, 59).

### 1.7 Rationale

Urgent research is required to address the inequitable rate at which Aboriginal children are being placed into out-of-home care compared with non-Aboriginal children (12). Effective collaboration between sectors is critical to improving the health of Aboriginal people and populations through effective service provision (60). Therefore, this study seeks to contribute to knowledge that aims to improve health outcomes for Aboriginal people by creating an understanding of how intersectoral collaboration can be strengthened. Strengthening collaboration between sectors has been found to improve the quality of care for families, children and kinship carers involved, or at-risk of becoming involved, with the child protection system (36, 37). Considering the WA Government’s announcement of the Aboriginal Community Controlled Organisation Strategy 2022–2032, the findings from this study will be useful for informing government policy action (30). At a national level, this study interrogates the Closing the Gap priority reform areas for joint national action (57). While this research is relevant for all four priority reform areas, priority reform one (formal partnerships and shared decision making) and priority reform two (building the Community-Controlled Sector) are particularly relevant to this study (57). This study will also inform priority focus area 2 in the National Child Protection Framework, which is interested in ‘addressing the over-representation of Aboriginal and Torres Strait Islander children in child protection systems’ (61).

### 1.8 Research questions

This study aims to better understand how the current system operates to provide recommendations for the strengthening of the out-of-home care system to improve health and wellbeing outcomes for Aboriginal people. Therefore, this research paper proposes the following questions:

1. ‘What are the views and narratives from Aboriginal primary healthcare staff in Western Australia about intersectoral collaboration challenges with regard to supporting families, children and kinship carers involved, or at risk of becoming involved, with the child protection system?’
2. ‘What are the views and narratives from Aboriginal primary healthcare staff in Western Australia about how the current system can be strengthened to improve intersectoral collaboration to better support families, children and kinship carers involved, or at risk of becoming involved, with the child protection system?’

## 2 Methods

### 2.1 Study design

This research utilised qualitative data collected largely by an Indigenous research team as part of the broader Indigenous Child Removals WA (I-CaRe WA) project, which is a mixed-methods study with overall aims of identifying factors to reduce the number of Aboriginal children in WA entering out-of-home care and methods to better support at-risk families, children in care and kinship carers. Seven WA ACCHOs were engaged in the I-CaRe WA study as research partners. The qualitative data set comprises 55 primary healthcare worker semi-structured interviews, four primary healthcare worker focus group discussions and 46 kinship care semi-structured interviews. This research project analysed the Aboriginal primary healthcare worker data (interviews and focus group discussions). The I-CaRe WA study was funded through the National Health and Medical Research Council (NHMRC).

### 2.2 Ethics approval

This research was approved by:

- Western Australian Aboriginal Health Ethics Committee (reference HREC 919)
- Curtin University Human Research Ethics Committee (reference HREC 2020-0428)
- University of Melbourne Central Human Research Ethics Committee (reference HREC 1956013)

### 2.3 Recruitment and sampling

Seven WA ACCHOs from the Perth metro, Kimberley, Pilbara, Midwest/Gascoyne and Southwest regions engaged in the study as research partners. All Aboriginal primary healthcare workers were recruited through convenience sampling at their workplace. The sample of Aboriginal primary healthcare workers included doctors, Aboriginal Health Workers, clinic nurses, receptionists, mental health practitioners, child health nurses and outreach workers. All prospective participants were provided with a Plain Language Statement (PLS) and consent form outlining the nature of the research, conditions of participation and details of interviewers (including their Aboriginal status). Participants were advised they could withdraw their participation at any time without providing a particular reason. Participants were then offered an opportunity to ask questions before providing written consent. As part of the consent process, participants were asked if they wanted a copy of their transcript. If a copy of the transcript was requested, it was sent via registered mail.

### 2.4 Data collection

Participant semi-structured one-on-one interviews and focus groups discussions were conducted by three university trained Aboriginal researchers from WA (authors two, four and five) with the assistance of one Aboriginal research assistant. Authors two and four are Aboriginal Chief Investigators on the I-CaRe project and formulated the interview guide with other project members. These data collection methods enabled the researchers to gain in-depth experiences from interviews and dynamic group perspectives from focus group discussions, which contributed to an overall information-rich qualitative data set (62, 63). Interview guides were structured around the key I-CaRe WA research aims and focused on participants’ experiences and perceptions. Primary healthcare worker interviews and focus group discussions were conducted in a private room on site at each of the ACCHOs. Interviews averaged 40 min and focus group discussions averaged 60 min. All participants were provided the option to debrief after each session and support services were made available throughout the research period and on a continuing basis (as required). Each session was audio recorded on digital recorders. Recordings were then transcribed verbatim by an independent confidential transcription service and then transcriptions were deidentified. Data were collected between 2018 and 2021.

### 2.5 Data analysis

The research team utilised NVivo 12 software to analyse transcriptions from interviews and focus group discussions. Guided by Constructivist Grounded Theory (CGT), data were independently coded by authors one, two and three using principles of Reflexive Thematic Analysis (RTA), which is an approach to analysing qualitative data that considers participant’s perceptions, views and experiences, when answering research questions related to a particular phenomenon (64, 65). Authors two and three coded a sample of interviews to develop inter-coder reliability. This analysis approach was selected so that the findings would centER the voices of Aboriginal and non-Aboriginal primary healthcare workers by allowing themes to emerge from participant experiences rather than pre-existing frameworks or theories (64–66). The first author of this study is a non-Aboriginal person. Aboriginal researchers provided oversight and input throughout this study. Data was stored on university shared protected drives and consensus was achieved through ongoing meetings with authors one, two and three and confirmed at project level meetings.

## 3 Results

**Figure 1:**
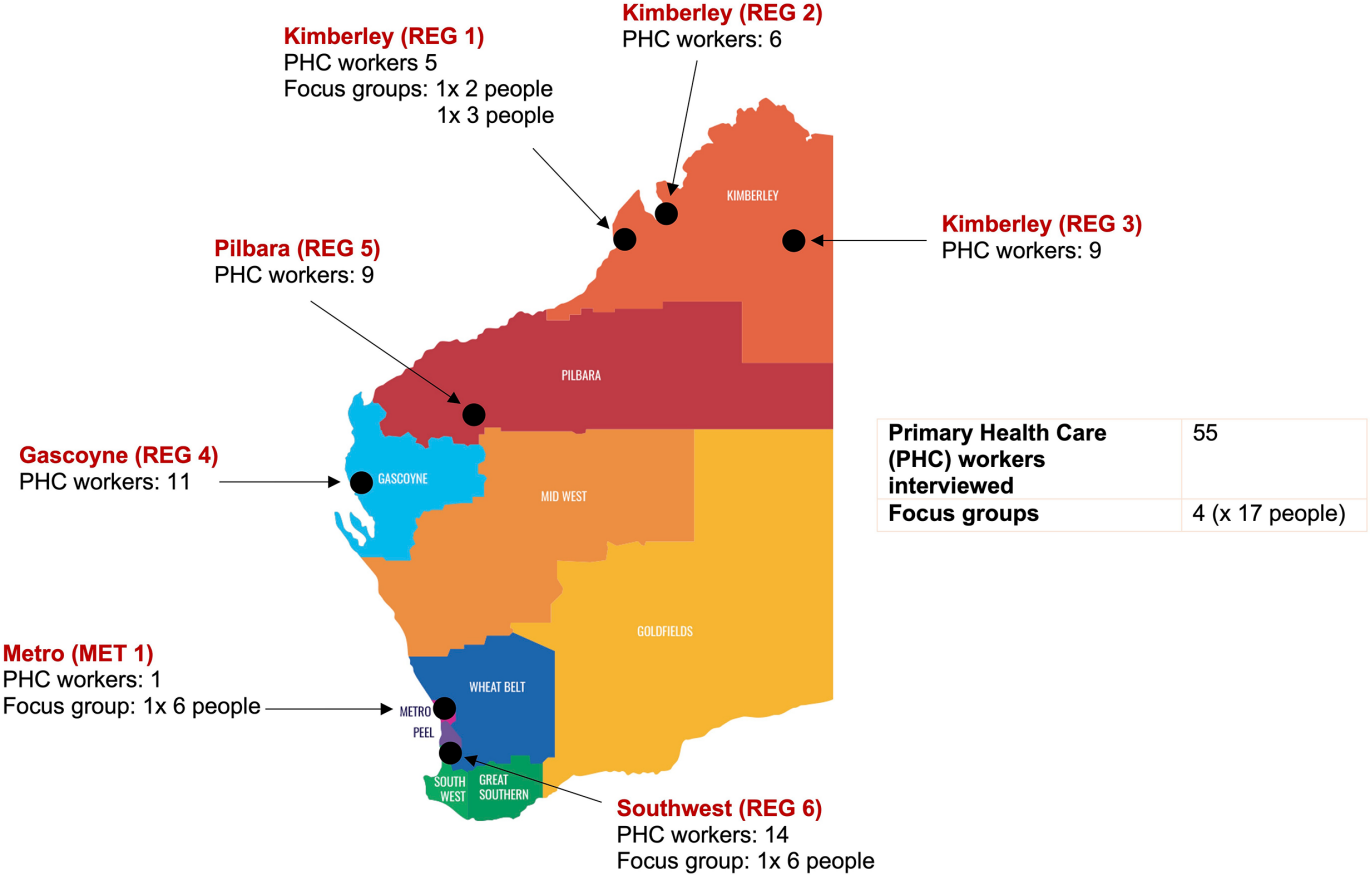
Map of Western Australia with data collection locations.

Aboriginal primary healthcare workers were a mix of Aboriginal and non-Aboriginal staff. Table 1 and Figure 1 illustrate the data set and the locations of the participants. The key sectors identified by Aboriginal primary healthcare workers included health, social services, education, and housing across government, non-government, and community organisations.

**Table 1:** Data collected per site.

Region	Code	Number of interviews	Number of focus groups
Metro	MET 1	1	1 (1×6 people)
Kimberley	REG 1	5	2 (1×2 people, 1×3 people)
Kimberley	REG 2	6	0
Kimberley	REG 3	9	0
Gascoyne	REG 4	11	0
Pilbara	REG 5	9	0
Southwest	REG 6	14	1 (1×6 people)

### 3.1 Intersectoral collaboration challenges

#### 3.1.1 Communication: information sharing

One of the key challenges raised across all ACCHOs related to communication difficulties. Numerous participants highlighted that communication with government social welfare services was challenging because of information sharing limitations. Although participants were unclear on exactly why this was the case, common explanations were lack of processes, confidentiality and privacy legislation.

*“I really do believe that AMSs [Aboriginal Medical Services, another name for ACCHOs] need to be involved in the conversation. You have - sometimes about the relationships you have and how you - how data is able to be allowed to pass from government organisations to AMSs and how that - how those relationships are formalised because there's quite a lot of work there I think that needs to occur to support, like you say, those - whether it's kinship carers or other carers and actually making sure that they're appropriately supported…”* (REG 6:10)

A number of participants detailed their view that information sharing between their services and government agencies was not reciprocal. Participants expressed their view that government departments often requested information but were hesitant to do the same in return. One participant detailed an example of a family support meeting, where different agencies across sectors were asked to come together and share information.

*“I find it difficult when it comes to - when they want to discuss about a family and the children, but that will get all the agencies to work together but they won't share the information out of it.”* (REG 3:2)

Several participants detailed how lack of information sharing between sectors meant that they were often provided with insufficient handovers when a client was referred to their service. Some participants highlighted how this affected their ability to provide an efficient service, while others highlighted risks associated with communication challenges. One participant reflected on a particular experience, when a child in their care was hospitalized for asthma yet this was not detailed in their handover from child protection services.

*“We didn't know that he was a severe asthmatic, he didn't have any medication. We went to check on him and saw that he was pretty sick, so we could end up with this little boy woke up in the morning and he’s gone. So, there’s not only that information given either.”* (REG_FG 6:1)

There was a sentiment across several participants that questioned how ACCHOs were supposed to succeed in intersectoral partnerships when they were often not provided with adequate information to do so effectively.

#### 3.1.2 Communication: interagency meetings

A recurrent example of intersectoral collaboration explored by participants was interagency meetings. While some participants reflected on the effectiveness of interagency meetings positively, others described experiences of unproductivity and difficulties in working relationships.

*“… there’s a child at risk meeting that I go to and they – we discuss families that either schools have referred or some of the other organisations have referred. Then you sit down and discuss the family and see what support they have. It’s definitely one – not against each other, but a lot of the time not working together.”* (REG 1:1)

The varied experiences of interagency meetings typically reflected the level of engagement and commitment different ACCHOs had to ensure strong working relationships with other agencies from health and non-health related sectors in the community and broader out-of-home care and kinship care system. Participants reflected on the difficulty of developing and sustaining strong working relationships, particularly considering the high staff turnover affecting the government child protection workforce. Some participants detailed that, while they viewed interagency meetings as important, sometimes they found it difficult to attend because of the time commitment required. Others reflected on difficulties in coordinating interagency meetings, and the need for more formalized invites and processes. While there were differences in experiences across ACCHOs, the general sentiment across most Aboriginal primary healthcare workers was that communication acted as a significant barrier to effective intersectoral collaboration.

#### 3.1.3 Relationship with the government sector: trust

While distrust of government services was highlighted by numerous participants as a barrier in terms of Aboriginal families engaging with non-Aboriginal services, many participants themselves highlighted distrust of government services as a challenge they experience within intersectoral partnerships.

*“I know our team has a really good relationship with Child Protection and Family Services (CPFS), but I'm still wary of them. I honestly, I've been to lots of signs of safety [meetings], and I still sit back, and I just watch, because I just feel like they're lions prowling in the background. I don't trust them whatsoever; I feel very unsafe around them. I don't know if that’s just the cultural thing for me, but I don't yeah, I'm not - I don't feel comfortable in their building. I don't know why it's just something.”* (REG_FG 6:1)

A significant number of participants were Aboriginal members local to the communities in which they were providing services. In addition, many of the participants had experiences of being kinship carers themselves.

*“I've also been a grandparent carer for about 13 kids, so lived experience and work.”* (REG_FG 6:1)

It was common for participants who did not detail direct personal involvement with child protection services to reflect on the experiences of their friends and family. Other participants provided their views on the broader system, discussing historical, and current, experiences of systemic racism from both professional and personal perspectives as drivers of mistrust. The recognition that Aboriginal primary healthcare workers themselves have shared identity and experiences with service users highlights an important consideration for intersectoral collaboration efforts between Aboriginal organisations and the government sector.

#### 3.1.4 Relationship with the government sector: the importance of ACCHOs being perceived by the Aboriginal community as independent

Several participants highlighted the importance of ACCHOs being perceived as trusted services that are independent of government services.

*“In the end, it's all there for the community, you know, and you want to work for the community and not for the departments. Family is everything, so I don't want to lose the trust. Lose the trust, that's it.”* (MET_FG 1:1)

This highlights an inherent challenge that these intersectoral partnerships face. Several participants provided accounts of the level of fear Aboriginal communities have around child protection services. Some participants explained many of the Aboriginal families they provided services to actively avoided government services because of a perception that if child protection services are present, they are only there to take children away. One participant explained that their ACCHO refused to have government child protection services personnel in their building because the presence of one person risks unravelling the trust their service had worked very hard to gain with their local community.

*“We won't have CPFS in the building at all, whatsoever, not even in the carpark. Because if our families saw that we had CPFS here, that could look very bad for us…we work very hard to gain trust with our families, and just that one person in the building could just ruin it all. So, we try not to do that.”* (REG_FG 6:1)

### 3.2 System strengthening opportunities to improve intersectoral collaboration

#### 3.2.1 Strengthening service resourcing and coverage: available services

Staff across all ACCHOs reported they were under-resourced for the amount of work they were doing. Several participants highlighted that family support services (that can help with prevention of child removal and reunification following removal) and kinship support services were significantly under-funded and under-resourced, which led to ACCHOs doing a lot of this work informally with little support. When asked about providing support to kinship carers, one Aboriginal primary healthcare worker provided the following account:

*“Well, realistically, there’s no organisation that gets funded to work in that space, it’s only because we’re connected to the community and we’re flexible that we’ll be able to maybe tap into their need if we have to. They come to us, and they ask us for help, and we’ll try and help them, but there’s no formal support in place, nothing at all for kinship carers. You really do need extra resources because otherwise it’s impacting on our resources and [draining] our resources really.”* (REG 3:5)

While lack of available service providers meant ACCHOs were taking on the burden of gaps in service provision, the lack of funding ACCHOs received for these services meant their services were stretched. Several participants highlighted the added difficulties of adequately supporting non-metropolitan clients seeking services, including family support, crisis accommodation and alcohol and drug support.

*“Because of the lack of support for the parents, like single dads, single mums, people with issues, like drug and alcohol issues, homelessness, family support, there are a lack of services to support people.”* (REG 3:3)

Family and kinship support services were highlighted as a key area for system strengthening. While many Aboriginal primary healthcare workers viewed support for formal kinship carers as under-resourced and under-funded, informal kinship carers were often not accounted for in the kinship care system. One participant reflected on their experience with an informal kinship carer:

*“Sometimes I see the grannies who have taken on the kids and sometimes it's not with Department of Child Protection (DCP) [now called the Department of Communities, responsible for child protection] help and I'm kind of interested in that because I often ask, 'are you getting any help with money or anything' and often, they're not, they're not getting anything. They're just doing it.”* (REG 6:5)

The high number of informal kinship carers that do not have access to formal support is a critical area for system strengthening as this gap limits carers’ ability to receive respite, focus on their own health and be appropriately supported when looking after children in their care. Another participant reflected on their experience trying to get support for a child not registered with child protection services:

*“One time I took a lady, she didn’t have any food for her kids, I took her around to six other services in town and not one could help her… One of them was DCP and they said, oh they have to be registered, they have to be under DCP in order to give them a food voucher.”* (REG 3:3)

#### 3.2.2 Strengthening service resourcing and coverage: addressing high program turnover

The rate of program turnover was discussed as a key area for system strengthening, with participants emphasising that programs were often not provided enough time to become established in communities and were sometimes shut down before they had an opportunity to create successful outcomes.

*“I think what’s really frustrating is I’ve been told that Aboriginal people have the highest amount of pilot programs, and they just get – and if they don’t meet the KPIs, they get cut. But it doesn’t mean that they’re not working, and if you keep having programs that keep getting turned over, people aren’t going to trust in these services, because they’re not going to last.”* (REG 4:6)

While the high turnover of programs has implications for building trust in the community, this also creates difficulties for ACCHOs and other service providers in establishing secure external referral pathways for clients seeking access to culturally appropriate services.

*“Then you might think there are services there, but then they’ve folded and then you’ve got to try and find something else. So, yeah. I think just the biggest gap that we have at the moment is there's no culturally appropriate service for Indigenous youth…”* (REG 6:11)

One participant explained the provision of short-term annual service contracts was common and contributed to the disorganized and unstable broader support service environment that made it difficult for ACCHOs to find support for clients outside of their own services. While under servicing of programs in communities was raised by numerous participants, surprisingly overservicing was reported as an issue in some areas too. One participant detailed their experience:

*“I feel like I guess one of the biggest problems is that we have a lot of organisations that do the same job. Or have the same type of roles. So, then you've got one client linked into like six different services. Then it starts to get confusing because you’re going to this person, this person, this person. You know talking about all the same things. Or you've got like three services working on the same thing for this young person. Then things are just getting lost in translation. I find that happens a lot, yeah.”* (REG_FG 1:2)

#### 3.2.3 Shift in system approach: Aboriginal-led care

A significant number of participants highlighted the need for other sectors to adopt a model of care that emphasised Aboriginal-led care, including Aboriginal people in leadership positions. The need for more Aboriginal Health Workers, social workers, mental health practitioners, government case workers, among others, was highlighted numerous times as an enabler to intersectoral partnerships and health-seeking behaviour of families and kinship carers involved with the child protection system.

*“I think having more Aboriginal people trained in positions to help people – in these big positions – so they don’t feel so lonely in the process. Because there are so many different people that they have to explain the same story to…”* (REG 6:4)

It was common for Aboriginal primary healthcare workers to relay the experiences of service users expressing their frustration with having to retell their stories numerous times when becoming involved with the child protection system.

*“The other thing that we're finding is Aboriginal people or First Nations people find it incredibly hard to tell their story again to a stranger, someone who hasn’t got the same Aboriginal terms of reference and the cultural background or some insight.”* (REG 4:1)

From the perspective of Aboriginal primary healthcare workers, lack of Aboriginal workers and leadership across health and non-health-related agencies and sectors meant it was difficult for Aboriginal community members to relay their circumstances or needs to non-Aboriginal workers or organisations that did not have the same Aboriginal terms of reference or cultural understanding derived from lived experience. There was a perception among many Aboriginal primary healthcare workers that, because of this, often the needs of service users were misinterpreted or overlooked. Numerous Aboriginal primary healthcare workers provided the view that the system needed reform to account for Aboriginal models of care and not solely Western approaches.

#### 3.2.4 Shift in system approach: aligning approaches

Several participants believed that different sectors were taking different approaches to supporting families, children, and kinship carers involved, or at risk of becoming involved, with the child protection system. When discussing intersectoral collaboration with the government sector, one Aboriginal primary healthcare worker relayed the below:

*“I feel that they should strengthen that working relationship with our organisations because they come from a crisis driven approach, whereas our approach is about prevention and early intervention. So, we need to find a balance to be able to match up and complement each other, but they need to really show a respect to our Aboriginal organisations because we are the ones that are grounded in the community.”* (REG 3:5)

The reported differences in approaches between sectors indicate that different agencies are working within different models of care and value systems. Families at risk of having their children removed are receiving, or being directed to, family support services from ACCHOs (and often without ACCHOs receiving funding). However, the system as a whole in participants’ views does not place as much emphasis on support and prevention outside of the community service level. Several participants advocated for more systemic emphasis on early intervention and support services to (a) reduce the rate of Aboriginal children entering out-of-home care and, (b) reduce the pressure on the finite number of kinship carers. The needs of families seeking reunification and formal/informal kinship carers requesting support were also commonly referred to by Aboriginal primary healthcare workers reflecting on how the system outside of their organisations was misaligned and not necessarily working within the same model of care.

## 4 Discussion

This research explored the experiences of Aboriginal primary healthcare workers in relation to intersectoral collaboration challenges and opportunities to better support families, children, and kinship carers involved, or at risk of becoming involved, with the child protection system. Through our analysis, we identified the following intersectoral collaboration challenges: communication (information sharing and interagency meetings); and the relationship with the government sector (trust and perception of ACCHO independence). Our analysis also identified the following key strengthening areas to improve intersectoral collaboration: strengthening service resourcing and coverage (improving availability of services and addressing high program turnover); and a shift in approach (Aboriginal-led care and aligning approaches).

One of the key challenges represented across all sites related to communication, with a lack of formalized communication processes contributing to information sharing limitations. Experiences of interagency meetings produced varied positive and negative accounts, which often reflected the strength of existing relationships rather than consistent, formalized approaches to meetings. Effective communication is perhaps one of the most universal and integral themes discussed in the literature around intersectoral collaboration. Communication is essential to establish trust and facilitate collaboration in intersectoral partnerships (67). Similar to Osborn et al. (53), a lack of structure around communication processes was found to contribute to systemic inefficiencies that were detrimental to intersectoral collaboration. This is consistent with Jones et al.’s (54) recommendations for system improvement, including better communication between sectors in a WA Aboriginal maternal and child health setting.

Relationships with the government sector were also highlighted as a key challenge. Trust was an issue in intersectoral partnerships given that many Aboriginal staff members had shared identity and experiences with service users. Aboriginal primary healthcare workers highlighted the importance of ACCHOs being perceived as independent services by Aboriginal communities because of the fear community members had around child protection services. Tension in the intersectoral partnership between ACCHOs and the government sector highlighted the intergenerational effects of colonisation, including historical and modern experiences of discrimination (1, 2). This is consistent with findings from other Indigenous health settings (68–71). In a broader sense, these challenges can be related to the external influences identified by Alhassan et al. (40), which described how historical and political contexts can shape the effectiveness of intersectoral collaboration.

While mistrust toward government services among Aboriginal populations in healthcare settings has been widely documented, the intersection of professional and personal mistrust among Aboriginal workers has not been explored. The reported lack of trust (which feeds into the importance of perceptions of ACCHO independence) highlights a significant implication for effective collaboration in this setting. This is because trust is a key pillar of intersectoral collaboration in practice (37). However, some researchers have found that, in the absence of trust, effective intersectoral collaboration is still possible in partnerships that are managed well and have well set out processes, goals, and conditions of collaboration (72, 73). Addressing the high staff turnover affecting the government child protection workforce would be a good starting point to facilitate effective communication pathways and improve trust in these partnerships (37).

Strengthening service resourcing and coverage was highlighted as a key strengthening opportunity to improve collaboration. Lack of available services, particularly in the family support and kinship support space, led to Aboriginal primary healthcare workers feeling unsupported in their intersectoral partnerships because they were taking on the burden of gaps in service provision without appropriate resources and funding. The high rate of program turnover in the broader support system provided insight into the constantly changing service environment. These findings demonstrate how the current service environment is incompatible with forming a structured process-based model of care (39). A key avenue to strengthen intersectoral collaboration is to have greater attention to intersectoral systems integration (52, 74). Better intersectoral systems integration improves the quality, continuity, efficiency, and effectiveness of care by making the broader system easier to navigate for providers and service users (52). However, lack of services, poor access to services, program turnover, among others, have significant implications for intersectoral collaboration (34). In Lopez-Carmen et al.’s (52) study, these factors were found to be barriers to achieving intersectoral service integration.

Greater attention on integrating services is also an enabler to ensuring primary health providers are directing people to culturally safe external services (52). This resonates with our findings, whereby Aboriginal primary health workers described difficulties in establishing culturally secure referral pathways in the current service environment. Participants described how this issue was more apparent in non-metropolitan areas and particularly when trying to connect people to external family and kinship support services. Informal kinship carers were particularly disadvantaged and unaccounted for in the broader support system. While there are no studies that explore the specifics of this experience, the under-resourcing of ACCOs is well documented (24, 75).

A call for a shift in approach was raised as a key strengthening opportunity. Participants advocated for more emphasis on Aboriginal-led care, including Aboriginal people in cross-sectoral leadership positions, to promote a model of care that better understood and met the needs of Aboriginal people. This reflected a call for self-determination and Aboriginal people having more decision-making power (76–79). Increasing Aboriginal peoples’ participation and leadership in the workforce is known to improve outcomes for Aboriginal populations (80). This can be addressed by improving pathways for Aboriginal people to receive training and qualifications, improve workforce skills and access leadership capacity development opportunities (81, 82). Other researchers have pointed toward Aboriginal models of care being more impactful by virtue of their empowerment-centric values, which typically focus on increasing the practical knowledge and skills of individuals and communities to improve health outcomes (48). A refocus on Aboriginal models of care and wellbeing has the potential to inform meaningful action to complex problems that intersect across multiple sectors, including health, social services, education, and housing (48).

The need for different sectors to align their approaches was considered an inherent strengthening area. There was a perception that different sectors were working within different care models with different priorities. Experiences of misaligned approaches are in line with ACCHOs holding an emphasis on holistic models of care compared to ‘traditional’ Western models (31). While Australia has historically focused on child protection orientations to child protection (‘identifying children at risk of abuse or neglect’) there has been rising demand for jurisdictions to adopt family support orientations (‘strengthening the care and capacities of parents’) in recent years (83, 84). Findings highlighted the complementary nature of ACCHO holistic models of care with family support approaches and provided insight into the perception of cross-sectoral misalignment (83, 85, 86). The Family Matters report findings, detailing WA having the lowest proportional investment in early intervention and family support services in Australia, further contextualizes these participant perceptions (22, 24). While reviews of the implementation of the ATSICPP have acknowledged the need to decrease crisis-based approaches to Aboriginal child protection, this study has found that there is a considerable amount of change required to transition to family support and prevention approaches (87).

Many of our research findings, including themes discussing the importance of effective communication, strong intersectoral partnerships built on trust, availability and coverage of services, adequate funding and having a shared vision and approach were consistent with findings across a range of different contexts (36, 37, 39–44). This demonstrates alignment with Kuruvilla et al.’s (46) findings, which found similarities in how sectors collaborated between different research contexts. While this study’s findings demonstrate some alignment with other studies on a broader level, context matters and future research should remain cautious about the transferability of these results and critical of how a particular research context might influence intersectoral collaboration (88). This is because varying structural, environmental, historical, and social mechanisms shape and influence how different sectors collaborate (89).

This study adds to the limited body of research on intersectoral collaboration in primary healthcare settings. Importantly, this study adds to the body of research specific to Australian Aboriginal health contexts and addresses a significant research gap concerning how different sectors collaborate in the context of Aboriginal child removals and kinship care. Overall, the research findings highlighted the need to review the current model of care to address some of the intersectoral collaboration challenges and opportunities raised by Aboriginal primary healthcare workers. More formalized structures and modes of collaborating are critical to ensure ACCHOs can succeed in their intersectoral partnerships to provide efficient and effective care (34, 53, 54). Considering the WA Government relies on intersectoral partnerships with ACCHOs to provide culturally secure services to Aboriginal people, addressing intersectoral collaboration challenges and opportunities is critical to improve the out-of-home care and kinship care system. This is relevant in the context of the WA Government’s commitment to better support ACCHOs, as detailed in the Aboriginal Community Controlled Organisation Strategy 2022–2032 (30). Findings from this research also provide evidence on how WA is tracking with the Closing the Gap strategy and National Child Protection Framework (57, 61).

### 4.1 Recommendations for future directions

There is a need to gain an in-depth understanding of how the current child protection and kinship care system in WA operates (formally and informally), how different sectors can improve the way they collaborate, and identify future collaboration opportunities. Reviewing the current model of care, including the roles of different sectors, would be a beneficial starting point to inform system reform. Working toward strengthening intersectoral systems integration would be a key reform area, to improve the efficiency of the out-of-home care and kinship care system and identify gaps in the service environment (52). Recent evidence highlighting the important role of the Indigenous Patient Navigator (IPN) would also be another beneficial avenue to explore (90). Implementing an IPN would address some of the issues raised by Aboriginal primary healthcare workers around the difficulty of navigating the system for service users requiring culturally secure services (90). An IPN would also provide ACCHOs and service users with a structure to better navigate the out-of-home and kinship care system and service environment (90). Interrogating the current system and adopting innovative ways to strengthen and reform its mechanisms is a necessary step to better meet the needs of Aboriginal people, reduce the rate of Aboriginal children entering out-of-home care and better support kinship carers.

### 4.2 Strengths and limitations

This study is the first that explores intersectoral collaboration in the context of Aboriginal child removals and kinship care in WA. Aboriginal researchers led this study which was beneficial in ensuring cultural safety at all stages of the research process. This was particularly important during the collection of data on the sensitive topic of Aboriginal children in out-of-home care. The research is enriched by the voices and perspectives of Aboriginal primary healthcare workers from ACCHOs located in regional, remote, and urban areas in WA. This study provides a good foundation for future research, which is important considering the lack of evidence in this area. Because this research was focused on child protection and kinship care, a majority of participant perspectives were in relation to intersectoral collaboration between Aboriginal organisations and government child protection services. Because of this, we have been limited in our ability to explore collaboration with other sectors including education and housing. A broader mix of perspectives from stakeholders from other sectors, including government, other non-government organisations, and community stakeholders, would have provided richer in-depth data.

### 4.3 Conclusion

The overrepresentation of Aboriginal children in out-of-home care is a significant public health issue. While kinship care is the preferred out-of-home care placement option for Aboriginal children that are unable to live with their parents, kinship care is undervalued and under-funded in the child protection system. Therefore, this study sought to find methods to improve outcomes for Aboriginal people by contributing toward an understanding of how different sectors are collaborating in the context of the child protection and kinship care system. This study suggests strengthening intersectoral collaboration is a step in the right direction to improve how the child protection and kinship care system operates. Key themes identified by participants, including communication and issues in the relationship with the government sector, present challenges for ACCHOs. Improving service resourcing and coverage and shifting the approach of the broader child protection and kinship care system, were identified as avenues to strengthen collaboration between sectors. It is clear that the current system needs to be further reviewed to understand how to better meet the needs of Aboriginal people and populations. While improving the way in which different sectors collaborate is important to review, the deficiencies in the current system are complex and transcend issues with intersectoral collaboration. Ultimately, the pathway to improve the current system requires an intersectoral effort to achieve meaningful action and prevent further harm from what some Aboriginal community members are calling ‘another stolen generation’ (10).

## Data availability statement

The datasets presented in this article are not readily available because the dataset is only available to the research team for analysis and publication. Requests to access the datasets should be directed to JJ, j.jones@curtin.edu.au.

## Ethics statement

The studies involving humans were approved by the Western Australian Aboriginal Health Ethics Committee (reference HREC 919), Curtin University Human Research Ethics Committee (reference HREC 2020-0428), and the University of Melbourne Central Human Research Ethics Committee (reference HREC 1956013). The studies were conducted in accordance with the local legislation and institutional requirements. The participants provided their written informed consent to participate in this study.

## Author contributions

SM: Writing – original draft, Writing – review & editing, Formal analysis, Investigation, Methodology, Conceptualization. JJ: Writing – review & editing, Conceptualization, Data curation, Formal analysis, Investigation, Methodology, Supervision, Writing – original draft. RC: Writing – review & editing, Conceptualization, Data curation, Formal analysis, Investigation, Methodology, Supervision, Writing – original draft. RW: Writing – review & editing, Data curation, Methodology. CG: Data curation, Writing – review & editing, Methodology. AG: Writing – review & editing. MO'D: Writing – review & editing. DM: Writing – review & editing. BM: Writing – review & editing. SE: Writing – review & editing.
